# Effect of direct therapeutic ultrasound exposure of ovaries on histopathology, inflammatory response, and oxidative stress in dogs

**DOI:** 10.1186/s12917-023-03657-6

**Published:** 2023-07-20

**Authors:** Arian Rajabi, Asghar Mogheiseh, Saeed Nazifi, MohammadSaeed Ahrari-Khafi, AmirReza Dehghanian, Nasser Vesal, Amin Bigham-Sadegh

**Affiliations:** 1 Department of Clinical Sciences, School of Veterinary Medicine, Shiraz University, Shiraz, Fars, Iran; 2 Department of Pathobiology, School of Medicine, Shiraz University of Medical Sciences, Shiraz, Fars, Iran

**Keywords:** Cytokines, Laparotomy, Neutralization, Primordial follicles, Ultrasound

## Abstract

**Background:**

This research was designed to evaluate the effects of therapeutic ultrasound waves on ovarian germinal tissue and inflammatory cytokines (interleukin-6 (IL-6), IL1β, tumor necrosis factor-α (TNF-α)), acute phase proteins (serum amyloid A (SAA), C reactive protein (CRP)) and oxidative stress (total antioxidant capacity (TAC), and malondialdehyde (MDA)) in dogs. Twenty-six clinically healthy adult mix-breed female dogs were aligned into three groups. Laparotomy was performed in control (n = 6) and treatment (T5, n = 10; T10, n = 10) groups. The ultrasonic exposure of ovaries in treatment groups was performed during laparotomy by round motions of the therapeutic ultrasonic transducer on both ovaries (1 MHz frequency, 1.5 W⁄cm^2^) for 5 min in the T5 group and for 10 min in the T10 group. Blood samples were collected from the jugular vein into a plain glass tube on days 0 (before laparotomy), 3, 6, and 9 after surgery. All control and treatment groups’ dogs were ovariectomized for histological evaluation on day 60 after laparotomy or laparotomy + ultrasound exposure.

**Results:**

Direct exposure of ovaries with therapeutic ultrasound waves induced inflammation and oxidative stress comparison with the control group. Histopathological evaluation of treated ovaries with ultrasound waves indicated a decreased number of primordial follicles (ovarian reserve) and oocyte preservation scores compared with ovaries in the control group.

**Conclusions:**

These changes may cause subfertility in the long term. It seems that inflammatory response and oxidative stress are factors in the permanent damage of ovarian tissue.

## Background

Therapeutic ultrasound waves with different intensities and frequencies can cause damage and necrosis in the target tissues. These findings have been proven in male rats [1], ram [2], monkeys [3], and dogs [4–7]. Due to germ cell apoptosis, local testicular thermal treatment can induce reversible oligospermia or azoospermia in monkeys. Only one exposure of the testicles of monkeys or rats at 43* °C* caused reversible harm to the seminiferous epithelium. Regional heating of monkey testicles to 43* °C* water for two consecutive days (30 min per day) has shown that the quantity of sperm in the seminal fluid has dropped up to 80% at 28 days and is revocable [8]. Therapeutic ultrasound affects the tissue, mainly due to its heating effect due to tissue absorption of ultrasonic waves. The actual mechanism of the destructive ability of the applied ultrasonic waves is unknown. Therapeutic ultrasound waves have caused damage to sperms in in vitro conditions, and exposure to these waves in vivo has caused a decrease in the quality of sperms and irreversible histopathological changes in the sperm-producing tissues of the testicles. This method does not have the disadvantages of surgical methods, including bleeding and side effects after surgery and anesthesia. It has a permanent effect on sterilization in dogs and rats at the dosages used [1, 6, 7].

After any tissue injury, acute phase response occurs and is characterized by several different systemic effects, including changes in the concentrations of blood proteins called acute phase proteins (APPs). Some of the APPs decrease in concentration (negative APPs; albumin or transferring), and others of which increase in concentration (positive APPs; serum amyloid A (SAA), C reactive protein (CRP), haptoglobin (Hp), alpha-1-acid glycoprotein (AGP), ceruloplasmin (Cp), and fibrinogen). Production and response of APPs vary depending on the species. For example, in the dog, a strong response occurs with CRP. Major APPs (CRP and SAA) usually have an early and high rise in concentration and a very rapid decline in dogs. Most positive APPs are glycoproteins synthesized mainly by hepatocytes upon stimulation by proinflammatory cytokines and released into the bloodstream [9]. The main proinflammatory cytokines are interleukin (IL)-6, IL-1, and tumor necrosis factor-α (TNF-α). Serum levels of IL-6 markedly increase during an acute phase response in dogs. Cytokine assays could be used for quantifying the induced systemic response to infection or inflammation [10]. The acute phase response only lasts a few days; however, increasing APPs also have been described in chronic inflammation. In these cases, an aberrant continuation of some aspects of the acute phase response may contribute to the underlying tissue damage [11]. Exposure of dog testes with ultrasonic waves induces systemic acute phase response. It significantly increases the concentrations of CRP, SAA, and AGP in peripheral blood on days 3, 5, and 7 compared to those of the control group [12].

Ultrasonic waves can induce oxidative stress using the thermal effects in dog testes [12]. Oxidative stress is caused by an imbalance between the production and accumulation of reactive oxygen species (ROSs). The result of the reactions of ROSs with biomolecules is the formation of substances that can be used as markers of oxidative damage, such as malondialdehyde (MDA) [13]. Antioxidants are the first line of choice to take care of stress. Endogenous antioxidant defenses include a network of compartmentalized antioxidant enzymatic and non-enzymatic molecules usually distributed within the cytoplasm and various cell organelles [14]. Total antioxidant power considers the cumulative effect of all antioxidants present in blood and body fluids. It is considered a valuable indicator of the body’s antioxidant status to counteract ROS –related oxidative damage [15].

Our previous study showed that exposing the ovaries to therapeutic ultrasound waves from the abdominal skin may affect the ovarian tissue. However, we did not reach a definitive conclusion [16]. The female dog’s systemic response to ultrasonic waves and the straight impact of therapeutic ultrasound waves on the ovarian tissue has not been investigated. Therefore, the current study aimed to investigate the impact of ultrasonic radiation on inflammatory responses and oxidative stress in the first week and ovarian tissue changes during two months after direct exposure to ultrasonic waves.

## Results

### IL6

Time, group, and interaction of group and time factors significantly affected the concentration of IL-6 during this study (p < 0.0001). IL6 serum concentration was not significantly different between T5, T10, and the control group before and on day 9 after treatment. On days 3 and 6 after treatment, IL6 serum concentration in T5 and T10 groups significantly increased compared with the control group (p < 0.0001). Also, the increase of IL6 serum concentration in treated group T10 was significant compared to treated group T5 (p < 0.002). All groups had no significant difference in IL6 serum concentration between day 0 and day 9. There was a significant difference in the increase of IL6 serum concentration on days 3 and 6 vs. day 0 (p < 0.02). There was a significant decrease in IL6 serum concentration on days 6 and 9 vs. day 3 and between day 9 vs. day 6 (P < 0.01; Table 1).

**Table 1:** The results (mean ± standard deviation) of measuring different factors, including inflammatory cytokines, acute phase proteins and total antioxidant capacity, and lipid peroxidation index in the serum of dogs in the control group (only laparotomy, n = 6), the 5 min treatment group, laparotomy and exposure of the ovaries with ultrasonic waves for 5 min (n = 10) and 10 min treatment group, laparotomy, and exposure of the ovaries with ultrasonic waves for 10 min (n = 7) in the days before surgery (day 0), and the days after surgery and exposing the ovaries to ultrasonic waves (days 3, 6 and 9)

Factor	Group	Day			
		0	3	6	9
**IL6 (ng/ml)**	**Control**	0.3 ± 0.04^ A^	0.66 ± 0.07^aB^	0.37 ± 0.06^aC^	0.29 ± 0.04^ A^
	**T5**	0.31 ± 0.04^ A^	1.55 ± 0.1^bB^	1.01 ± 0.06^bC^	0.31 ± 0.04^ A^
	**T10**	0.29 ± 0.05^ A^	1.63 ± 0.11^cB^	1.13 ± 0.06^bC^	0.29 ± 0.04^ A^
**TNFα (ng/ml)**	**Control**	1.04 ± 0.46^B^	2.32 ± 0.85^aA^	1.37 ± 0.54^aB^	1.03 ± 0.42^B^
	**T5**	0.97 ± 0.48^ A^	3.49 ± 0.44^bB^	2.18 ± 0.15^bC^	1.10 ± 0.47^ A^
	**T10**	1.08 ± 0.47^ A^	3.86 ± 0.41^bB^	2.29 ± 0.14^bC^	1.10 ± 0.43^ A^
**IL1β (pg/ml)**	**Control**	0.1 ± 0.02^B^	0.27 ± 0.06^aA^	0.14 ± 0.02^aB^	0.09 ± 0.03^B^
	**T5**	0.1 ± 0.02^ A^	0.54 ± 0.1^bB^	0.28 ± 0.08^bC^	0.1 ± 0.03^ A^
	**T10**	0.09 ± 0.02^ A^	0.57 ± 0.11^bB^	0.29 ± 0.08^bC^	0.11 ± 0.03^ A^
**SAA (mg/ml)**	**Control**	6.49 ± 0.77^B^	15.99 ± 1.85^aA^	8.22 ± 1.06^aB^	6.35 ± 0.77^B^
	**T5**	6.50 ± 0.74^ A^	41.01 ± 4.83^bB^	28.5 ± 4.40^bC^	6.54 ± 0.77^ A^
	**T10**	6.52 ± 0.67^ A^	45.38 ± 3.41^cB^	29.20 ± 3.60^bC^	6.59 ± 0.76^ A^
**CRP (µg/ml)**	**Control**	3.27 ± 0.45^B^	9.11 ± 1.32^aA^	4.07 ± 0.32^aB^	3.25 ± 0.47^B^
	**T5**	3.33 ± 0.43^ A^	30.56 ± 6.53^bB^	20.26 ± 3.55^bC^	5.29 ± 0.85^ A^
	**T10**	3.34 ± 0.37^ A^	36.66 ± 5.05^cB^	20.74 ± 4.39^bC^	5.35 ± 1.04^ A^
**TAC (mmol/l)**	**Control**	3.18 ± 1.05	2.79 ± 0.86	3.12 ± 0.85	3.18 ± 1.05
	**T5**	3.22 ± 1	2.70 ± 0.61	3.13 ± 0.82	3.20 ± 1.02
	**T10**	3.19 ± 0.85	2.54 ± 0.54	2.96 ± 0.70	3.19 ± 0.82
**MDA (µmol/l)**	**Control**	0.57 ± 0.03^B^	0.62 ± 0.05^aA^	0.57 ± 0.03^aB^	0.55 ± 0.02^B^
	**T5**	0.55 ± 0.03^ A^	0.77 ± 0.05^bB^	0.64 ± 0.05^bC^	0.55 ± 0.03^ A^
	**T10**	0.54 ± 0.02^ A^	0.81 ± 0.04^cB^	0.65 ± 0.04^bC^	0.54 ± 0.03^ A^

^ABC^ superscript letters indicate significant difference between days of study in each row

^abc^ superscript letter indicate significant difference between groups in each column

### TNFα

Time, group, and interaction of group and time factors significantly affected the concentration of TNFα during this study (p < 0.0001). TNFα serum concentration did not significantly differ between treatment and control groups on days 0 and 9. On days 3 and 6 after treatment, the increase of TNFα serum concentration in the T5 and T10 groups was statistically significant compared to the control group (p < 0.0002). In the control group, an increase in TNFα serum concentration on days 6 and 9 compared with day 0 and the decrease in TNFα serum concentration between days 9 and 6 were not significant. TNFα serum concentration was increased significantly on day 3 vs. day 0 in the control group (p < 0.0001). Also, TNFα serum concentration was significantly increased on days 6 and 9 vs. day 3 in the control group (p < 0.0004). There was no significant difference in TNFα serum concentration on day 0 vs. day 9 in T5 and T10 groups. There was a significant increase in TNFα serum concentration on days 3 and 6 vs. day 0 and a decrease of TNFα serum concentration on days 6 and 9 vs. day 3 in the T5 and T10 groups (p < 0.0001; Table 1).

### IL1β

Time, group, and interaction of group and time factors significantly affected the concentration of IL1β during this study (p < 0.0001). IL1β serum concentration did not significantly differ between T5, T10, and the control groups on days 0 and 9. IL1β serum concentration was significantly increased in T5 and T10 groups compared to the control group on days 3 and 6 (p < 0.0001). IL1β serum concentration was significantly increased on day 3 vs. day 0 in the control group (p < 0.0001). Also, IL1β serum concentration was significantly decreased on days 6 and 9 vs. day 3 in the control group (p < 0.0001). There was no significant difference in IL1β serum concentration on day 0 vs. 9 in T5 and T10 groups. There was a significant increase in IL1β serum concentration on days 3 and 6 vs. day 0 and a decrease in IL1β serum concentration on days 6 and 9 vs. days 3 in T5 and T10 groups (p < 0.0001; Table 1).

### SAA

Time, group, and interaction of group and time factors significantly affected the SAA concentration during this study (p < 0.0001). SAA serum concentration did not significantly differ between T5, T10, and control groups on days 0 and 9. SAA serum concentration was significantly increased in T5 and T10 groups in comparison to the control group on days 3 and 6 (p < 0.0001). SAA serum concentration in the control group was significantly increased on day 3 vs. day 0 (p < 0.0001). Also, SAA serum concentration was significantly decreased on days 6 and 9 vs. day 3 in the control group (p < 0.0001). There was no significant difference in SAA serum concentration on day 0 vs. day 9 in T5 and T10 groups. There was a significant increase in SAA serum concentration on days 3 and 6 vs. day 0 and a decrease in SAA serum concentration on days 6 and 9 vs. day 3 in the T5 and T10 groups (p < 0.0001; Table 1).

### CRP

Time, group, and interaction of group and time factors significantly affected the concentration of CRP during this study (p < 0.0001). CRP serum concentration did not significantly differ between T5, T10, and control groups on days 0 and 9. CRP serum concentration significantly increased in T5 and T10 groups compared to the control group on days 3 and 6 and in treated group T10 with treated group T5 on day 3 (p < 0.0001). CRP serum concentration was significantly increased on day 3 vs. day 0 in the control group (p < 0.0001). Also, CRP serum concentration was significantly decreased on days 6 and 9 vs. day 3 in the control group (p < 0.0001). There was no difference in CRP serum concentration on day 0 vs. day 9 in T5 and T10 groups. There was a significant increase in CRP serum concentration on days 3 and 6 vs. day 0 and a decrease in CRP serum concentration on days 6 and 9 vs. day 3 in the T5 and T10 groups (p < 0.0001; Table 1).

### TAC

Group, time, and interaction of group and time factors did not significantly affect the TAC concentration. TAC serum concentration was not significantly different between T5, T10, and control groups on days 0, 3, 6, and 9. There was no significant difference in TAC serum concentration between sampling days in each group (T5 and T10 treatments and control; Table 1).

### MDA

Time, group, and interaction of group and time factors significantly affected the concentration of MDA during this study (p < 0.0001). MDA serum concentration did not significantly differ between T5, T10, and control groups on days 0 and 9. MDA serum concentration was significantly increased in T5 and T10 groups in comparison to the control group on days 3 and 6 (p < 0.0001). Also, MDA serum concentration was significantly increased in treated group T10 compared to treated group T5 on day 3 (p < 0.02). MDA serum concentration was significantly increased on day 3 vs. day 0 in the control group (p < 0.0001). Also, MDA serum concentration was decreased on days 6 and 9 compared to day 3 in the control group (p < 0.0001). There was a significant increase in MDA serum concentration on days 3 and 6 vs. day 0 and a decrease in MDA serum concentration on days 6 and 9 vs. days 3 in the T5 and T10 groups (p < 0.0001; Table 1).

### Histopathological evaluation

The tissue necrosis score (TNS), oocyte preservation score (OPS), cortical thickness, and the number of primordial, primary, and secondary follicles of ovaries were compared between the treatment and control groups. The cortical thickness, mean number of primary and secondary follicles, and TNS were not significantly different between the treated (T5 and T10 groups) and control groups. However, there were fewer primordial follicles in the treated (T5 and T10 groups) than control and in the T10 group than T5 group (p < 0.0001). The OPS was fewer in treated group T5 than control (p < 0.03) and also was fewer in treated group T10 than control (p < 0.0004). The value of OPS was not different between the treated groups (T5 and T10 groups; Table 2).

**Table 2:** The results (mean ± standard deviation) and comparison of the histopathology evaluation of dog ovaries during two months after laparotomy of the control group (n = 6), the laparotomy group and exposure of the ovaries for 5 min with ultrasonic waves (n = 10), and the laparotomy group and exposure 10 min of ovaries with ultrasonic waves (n = 7)

Group	Average Cortical Thickness (mm)	Primary follicles/HPF	Secondary Follicles/HPF	Primordial follicles/HPF	Oocyte preservation score (OPS)	Tissue necrosis score (TNS)
**Control**	0.9 ± 0.19	2.83 ± 1.47	1.33 ± 0.65	9.83 ± 3.27^a^	9.67 ± 0.78^a^	0 ± 0
**T5**	0.78 ± 0.30	1.37 ± 0.76	1.37 ± 0.76	7.68 ± 3.15^b^	8.32 ± 1.20^b^	0.53 ± 2.29
**T10**	0.88 ± 0.25	1.67 ± 0.49	1.42 ± 0.51	4.92 ± 2.02^c^	7.5 ± 0.90^b^	0.83 ± 2.89

^abc^ superscript letter indicate significant difference between groups in each column

## Discussion

In our study, direct exposure of ovaries with therapeutic ultrasound waves induced inflammation and oxidative stress compared with the control (laparotomy) group. Histopathological evaluation of treated ovaries with therapeutic ultrasound waves indicated decreased primordial follicles (ovarian reserve) and oocyte preservation scores compared with controls (laparotomy).

In this study, laparotomy increased inflammatory cytokines and acute phase proteins in the first three days after surgery. These factors decreased during the sixth and ninth days after surgery and returned to the level of the day before surgery. Surgeries performed in dogs, including spaying surgeries, increase inflammatory cytokines and phase proteins during the first week after surgery [17, 18]. The duration of surgery, intra-operative manipulations, and drugs affect the duration and intensity of the inflammatory response [19]. Both ovariohysterectomy and ovariectomy caused major post-surgical changes in iron serum levels, CRP, and glucose. CRP is a significant acute phase protein in dogs, and a mild inflammatory response happens after ovariectomy and ovariohysterectomy [20]. Ovariohysterectomy is a spaying method commonly employed in female dogs. A surgical technique that interrupts tissue integrity induces a temporary local inflammatory reaction accompanied by a systemic response called an acute phase response [20, 21]. Inflammatory processes are subject to underlying ovarian pathology; pro-inflammatory conditions may negatively impact ovarian follicular dynamics if pushed. Aberrant inflammation can alter normal ovarian follicular dynamics, impairing oocyte quality, anovulation, and associated infertility. C-reactive protein is an acute-phase reactant produced by hepatocytes and is a marker for systemic inflammation. Serum CRP levels rise in response to increased TNF-α and IL-6 from macrophages and adipocytes, activating an inflammatory response through the complement system [22]. Low-grade, chronic inflammation impaired folliculogenesis [23]. ROS are important to ovulation, but in excess, they are cytotoxic to the cell and organelles. Without a responsive increase in antioxidants, oocytes are subsequently damaged or of poorer quality through the phenomenon known as lipotoxicity [24]. Early expression of these inflammatory markers may induce a sudden influx of leukocytes and, as a result, impair maturation and subsequent ovulation [25]. Additionally, inflammation has been associated with oxidative stress, and oxidative stress has been shown to induce inflammation, thus suggesting a perpetuating cycle.

In the present study, exposing the ovaries to therapeutic ultrasound waves for 5 or 10 min increased the intensity of the inflammatory response, especially in the first three days. Exposure of dogs’ testicles to ultrasonic waves induced an inflammatory response, and it seems that this inflammatory response plays a role in the permanent damage of testicular tissue [12].

The oxidative stress index (MDA concentration) increased in the laparotomy group. Increasing of MDA level in T5 and T10 treated groups was greater than in the laparotomy group. Oxidative stress destroys the tissue and can cause permanent damage and infertility in sensitive tissue such as the testis and ovary. Exposure of dogs’ testicles to ultrasonic waves has induced oxidative stress in the tissues and probably plays a role in their permanent damage and sterility [12]. Ultrasonic radiation is one of the safe methods to control fertility in male dogs. It was Fahim et al. who introduced ultrasound as a method of male fertility control for the first time in 1975. Leoci and co-workers (2015) exposed every dog testicle to therapeutical ultrasonography (1.5 W/cm^2^, 1 MHz) for 5 min every two days and for three therapies. Following two weeks, a notable decline was seen in the sperm count and testicle size of the dogs [7]. High-intensity ultrasonic radiation could trigger the oxidative stress and acute phase response of inflammation and create more injury to the testicle tissue in dogs. This was seen mostly in the first week following the testicles have been exposed to ultrasonic radiation [12]. It has been stated that radiofrequency electromagnetic wave exposure from cell phones can motivate oxidative stress, decline the action of antioxidant enzymes (glutathione peroxidase (GPx) and superoxide dismutase (SOD)), and raise the density of MDA in the sperm of male Wistar rats. It may be related to sterility and a change in reproductive function [26]. Tang et al. 2015 summarized the non-thermal effect of ultrasound (focused, pulsed ultrasound frequency 20 kHz-3 MHz) on sonomechanical, including acoustic radiation force, shockwave, and micro-jets induced by cavitation and sonochemical including inertial cavitation generated reactive oxygen species (ROS) and intracellular ROS release from mitochondrial damage. Their biological effects are cytoskeletal remodeling, cellular proliferation, protein synthesis modulation, enhanced gene transfection, induction of oxidative stress, and up-regulation of apoptotic genes [27].

Histopathological changes after direct exposing the ovaries to ultrasonic waves explain that the count of primordial follicles and the egg preservation score decreased. These changes can cause subfertility in the long term. The lower temperature of the follicular fluid prior to ovulation may be essential for normal oocyte development. Changes in temperature within the ovary at the ovulatory phase may be important for optimal nuclear cytoplasmic and membraneous maturation [28]. Local cooling of the female reproductive tract, especially of specific ovarian tissues, occurs close to ovulation, favoring male and female gamete maturation [29]. A 1 °C increase in average maximum temperature during the 90 days before ovarian reserve testing was associated with a -1.6% lower antral follicle counting [30]. In our preliminary study, the ovaries were exposed to ultrasonic waves through the skin, and limited and variable histopathological changes were found in the ovarian tissues of dogs [16]. Various protocols were tested for exposure of the testicles to ultrasonic waves, which gave relatively various outcomes. Indeed, the frequency and strength of waves, the time of exposure, frequency, and interval among exposures can affect the results [12]. Three treatments within 48 h intervals at 1 MHz, 1.5 W/cm2 for 5 min, lead to permanent testicular injury and azoospermia in the dog. No significant adverse effect was reported, and sterility occurred with testosterone concentration in the physiological range [7].

In ram, as the time and repeats of ultrasonication increase, the affected spermatozoa level increases, although the mitochondrial activity is reduced. The most effective ultrasonication related to the damaged spermatozoa and mitochondrial activity rate was seen in the protocol with an 8-repeating, 10-s duration group in Merino ram spermatozoa [2]. The combination of thermal and non-thermal (mechanical) impact of the ultrasonic waves causes an ion exchange between the fluid in the seminiferous tubules and the rete testis, creating an unsuitable condition for spermatogenesis that disrupt spermatogenesis [6].

Ultrasound treatment seems to be most efficient for males with smaller testes, suggesting that higher levels of exposure may be necessary to obtain fertility control in individuals and animals with larger testicular mass. It was verified in a non-human primate species with testes that compare in size with those of men that ultrasound treatment decreases sperm count and quality. This study gives evidence of the theory that testicular ultrasound exposure can be a practical tool for fertility control in humans [3]. Therapeutic ultrasound depleted growing germ cells from the testicle. They also noted that combining raised temperature, high power, and high frequency is essential to decreasing sperm number. Correctly combining these features is the hardest part of neutralization by ultrasound [1].

Therapeutic ultrasound therapy was found to be related to congenital deformities, and exposure to short waves was found to be associated with reduced birth weight and with enhanced danger for male infants. Women physiotherapists’ exposure to short-wave radiation during pregnancy could negatively affect pregnancy results, and short-wave use in pregnancy may be considered a potential risk for reproduction [31]. Exposure to short-waves and therapeutic ultrasound was associated with medically diagnosed spontaneous abortions happening after the tenth week of pregnancy but not with those happening before [32].

Controversially, trans-abdominal exposure of transplanted ovarian tissue with low-intensity pulsed ultrasound (5 min daily, 0.3 W/cm2, frequency of 3 MHz) in mice [33] and treatment of cases with premature ovarian failure in rats (frequency of 900 kHz, 90 W/cm^2^ for ten days) and mice (200 mW/cm2, frequency of 0.3 MHz) for 20 min, 15 consecutive days) [34, 35] could increase re-angiogenesis and promote ovarian follicular growth and some functions of ovaries.

It seems that direct exposure of ovaries with the therapeutic ultrasound has a limited effect on the ovarian tissue, especially since this treatment was not repeated like previous studies conducted on the testes of dogs and other species. The low number of animals in each group, without hormonal stimulation of ovaries and fertility tests following treatment, limited the results of this study.

## Conclusion

Therapeutic ultrasound (1 MHz frequency, 1.5 W⁄cm^2^) exposure to dog’s ovaries induced higher circulating inflammatory response and oxidative stress in T5 and T10 groups compared with the control (laparotomy) group. The number of primordial follicles and oocyte preservation scores decreased in ovaries treated with therapeutic ultrasound compared to ovaries in the control group. It is suggested that the direct exposure of ovaries with therapeutic ultrasound is studied using different protocols with higher frequency and power than this study and measuring anti-mullerian hormone as an index of ovarian follicular reserve following treatment.

### Methods

Experimental treatment regimens have been accomplished in compliance with Iran’s Animal Ethics system under the oversight of the Iran community to avoid harshness to Animals and the Shiraz University study board (IACUC no: 4687/63). The recommendations of the EU Committee Directive (2010/63/EU) of September 22, 2010, on animal welfare standards on a trial basis, were also monitored.

### Animals

Twenty-six clinically healthful mixed-breeds reproductively adult anestrous female dogs, aged approximately 1.96 ± 0.64 years old and weighing 17.55 ± 1.5 kg, were used for this research. The dogs were owned and maintained in the School of Veterinary Medicine Shiraz University, ovariectomized upon the study’s completion, and retained in a non-government shelter. Every dog has been fed with a commercial food dog (300 g/dog/day; Nutripet™; Behintash Co., Karaj, Iran) and was given ad libitum access to water. They were adapted to the new conditions during the first two weeks. All dogs were treated with anti-parasitic tablets (Fenbendazole, 150 mg; Pyrantel embonate, 144 mg; Praziquantel, 50 mg; Caniverm®, 0.7 mg/10 kg, PO). Overall, dog health was assessed daily after examining their body temperature, heart rate, respiratory rate, appetite, and attitude when feeding or cleaning their house. The state of pregnancy of dogs was checked with a trans-abdominal ultrasound before treatments. The phase of the estrous cycle was assessed with vaginal cytology.

### Experimental design

**Fig. 1:**
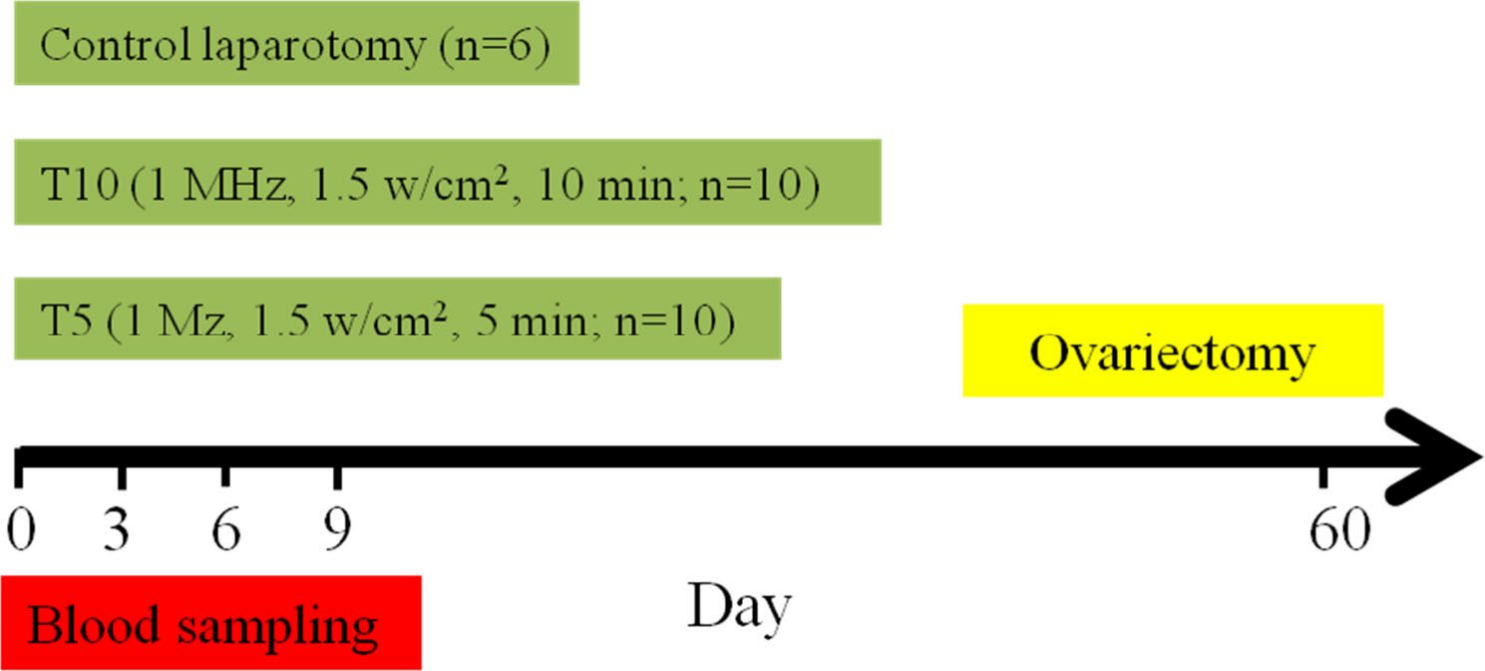
The dogs were aligned into three groups, a control (laparotomy) group (n = 6) and two treatment groups: 5 min treatment of ovaries with ultrasound wave during laparotomy (T5, n = 10), and 10 min treatment of ovaries with ultrasound wave during laparotomy (T10, n = 10)

**Fig. 2:**
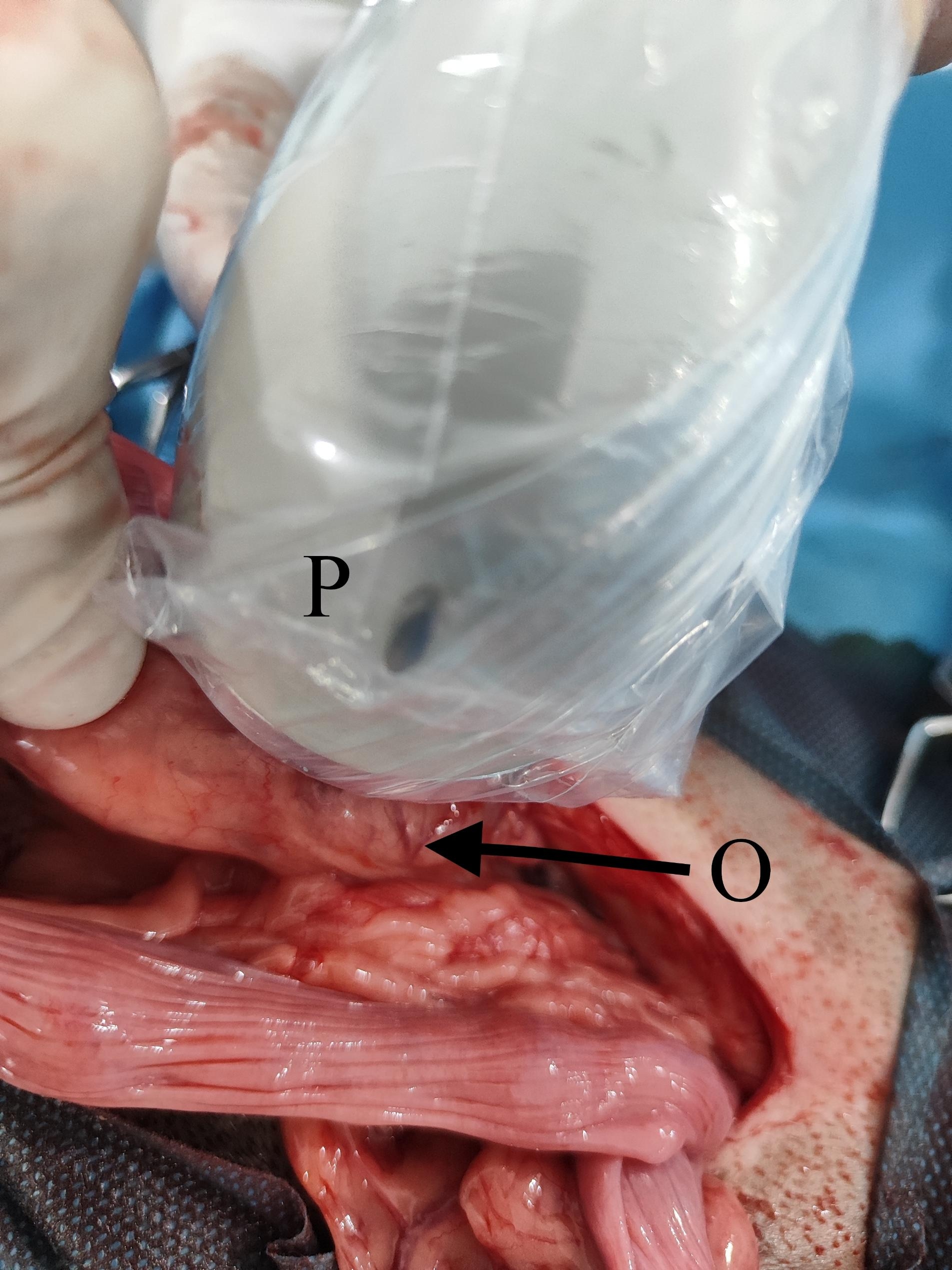
Dog ovaries were contacted directly with therapeutic ultrasound probe during laparotomy to evaluate the effect of this ultrasound waves (1 MHz frequency, 1.5 W⁄cm^2^) on histopathology of ovaries and systemic oxidative stress and inflammatory response. Therapeutic ultrasound probe was used by round motions on the ovaries. P: therapeutic ultrasound probe; O: ovary surrounded by ovarian bursa

The dogs were aligned into three groups, a control (laparotomy) group (n = 6) and two treatment groups: 5 min treatment of ovaries with therapeutic ultrasound wave during laparotomy (T5, n = 10), and 10 min treatment of ovaries with therapeutic ultrasound wave during laparotomy (T10, n = 10) (Fig. 1). Both ovaries are exposed without ultrasound treatment in the control group using a turned-off ultrasound probe. After laparotomy and exposing the ovaries in treatment groups, the therapeutic ultrasound (Unix U500, Iran) transducer was used by round motions on the ovaries (1 MHz frequency, 1.5 W⁄cm^2^) for 5 min in the T5 group and for 10 min in the T10 group (Fig. 2). Blood samples were obtained from the jugular vein on days 0 (before laparotomy), 3, 6, and 9 after surgery for evaluating inflammatory cytokines, acute phase proteins, and oxidative stress (IL-6, α-TNF, 1β-I, SAA, CRP, TAC, and MDA). The surgeon controlled the length of surgery similarly among the control (laparotomy) group and treatment (laparotomy + ultrasound exposure) groups.

### Laparotomy and ovariectomy

Laparotomy was performed under general anesthesia in control and treatment groups. Animals fasted for about 12 h before the surgery. The anesthesia protocol was chosen and applied under the supervision of the anesthetist. The dogs were pre-medicated via acepromazine (0.05 mg/kg, intra-muscular) and xylazine (0.5 mg/kg, intra-muscular). Anesthesia was induced using ketamine (5 mg/kg, Intra-Venous) and diazepam (0.25 mg/kg, Intra-Venous). After tracheal intubation, anesthesia was maintained with isoflurane (1.2%) sprayed into oxygen using intermittent positive-pressure ventilation. Tramadol (2 mg/kg; IM) and cefazolin (20 − 30 mg/kg; intra-muscular) were given at the onset and end of surgery. Ovariectomy was conducted for histological evaluation of ovaries on day 60 following ultrasound therapy.

### Histopathological evaluation

After the ovariectomy, the ovarian tissues were fixed in 10% phosphate-buffered formalin. After one day, a fresh solution was substituted for the formalin. Ovarian tissues were dried in an automated tissue transformer, and samples were embedded in paraffin. The Sects. (5–10 μm) was prepared with a microtome and stained with hematoxylin-eosin. The tissue structure was assessed using a light microscope, and various types of follicles were counted in three sections from each ovary. The ovaries were evaluated by a reproductive pathologist who was blind to the research groups. The count of follicles (primordial, primary, and secondary), tissue necrosis score (TNS), and oocyte preservation score (OPS) was identified in the ovary segments using the scoring method that has been used in human ovary research. The presence of total necrosis in each ovary was set by assessing the percent of necrosis using TNS per sample. The TNS is an observable necrosis assessment and presents the percentage of necrosis/infarction in ovary samples. TNS was rated from lowest (0) to highest [4] for characterizing necrotic transformation. The OPS was used to assess the level of histopathological injury. The OPS description was established to evaluate the structural preserving of follicles and oocytes and the embedding tissue micro-environment related to these structures. The intensity and progress patterns of tissue lesions were determined according to the magnitude of inflammatory alterations and tissue necrosis from one to nine [36]. Follicles have been divided into four classes: Primordial follicles (a layer of squamous granulosa cells nearby an oocyte), primary follicles (one layer of cuboidal granulosa cells nearby an oocyte), secondary follicles (a complete double layer of cuboidal granulosa cells nearby the oocyte), and antral follicles (multi laminar granulosa cells nearby the oocyte in the presence of an antrum). The follicles categorized in a section included an oocyte to inhibit the recounting the same follicles [37].

### Laboratory assays

Interleukin-6 (IL-6) was measured by a quantitative sandwich enzyme immunoassay using a commercial dog-specific competitive ELISA kit (CUSABIO, Shanghai, China, Code CSB-E11260c). The kit’s sensitivity was 0.39 pg/mL (the detection limit of the kit was 1.56 pg/mL-100 pg/mL). The intra-assay and inter-assay coefficient of variation of the interleukin-6 (IL-6) kit was CV < 8% and CV < 10%, respectively. C-reactive protein was measured using a canine solid- phase sandwich ELISA method (Shanghai Crystal Day Biotech Company, China; Catalog Number: CRP E 0124ca). CRP ELISA kit was specified with Intra-Assay CV < 8%, Inter-Assay CV < 10%, and sensitivity 7.8 pg/ml. Serum amyloid-A (SAA) was measured using a canine solid- phase sandwich ELISA method (Shanghai Crystal Day Biotech Company, China; Catalog Number: SAA E0125ca). SAA ELISA kit was specified with Intra-Assay CV < 8%, Inter-Assay CV < 10%, and sensitivity 0.156 pg/ ml). TNF-α was measured using a canine solid- phase sandwich ELISA method (Shanghai Crystal Day Biotech Company, China; Catalog Number: TNF E0025 ca.). TNF-α ELISA kit was specified with Intra-Assay CV < 8%, Inter-Assay CV < 10%, and sensitivity 0.01 ng/l). IL-1β was measured using a canine solid-phase sandwich ELISA method (Shanghai Crystal Day Biotech Company, China; Catalog Number: IL1B E0002ca). IL-1β ELISA kit was specified with Interleukin-1β Intra-Assay CV < 8%, Inter-Assay CV < 10%, and sensitivity 7.8 pg/ml) [38].

A commercial kit (ZellBio GmbH kit, Germany) determined the TAC level. The color product of the chromogenic substrate (tetramethyl benzidine) emerged at the ending phase. The color difference was calculated colorimetrically using a spectrophotometer (Jenway 6300 Spectrophotometer, UK) at 450 nm and represented as mmol/L. This method can determine TAC with 0.1 mM sensitivity (100 µmol/L). The intra- and inter-assay CVs were below 3.4% and 4.2%, respectively. An assay kit from ZellBio GmbH (Germany) measured MDA (µmol/L; Cat. no. ZB-MDA96A). In this kit, MDA is measured based on its reaction with thiobarbituric acid in an acidic condition and high temperature. The color complex was measured colorimetrically at 535 nm. The assay kit sensitivity was 0.1 µM (inter-assay CV: 5.8%) for MDA [39].

### Statistical analysis

The statistical analysis was performed using the Graph-Pad Prism 6 program utilizing the Two-way ANOVA repeated measures and paired tests for serum factors. The data were normally distributed. The One-way ANOVA repeated measures assessed statistical differences between treated and control groups. All results have been stated as mean ± SD. It was significant to be mean differences when there was a P-value of less than 0.05.

## Data Availability

The datasets generated and/or analyzed during the current study are available from the corresponding author on request.

## Abbreviations

IL-6: Interleukin-6
IL1β: interleukin-1β
TNF-α: tumor necrosis factor-α
SAA: serum amyloid A
CRP: C reactive protein
APPs: acute phase proteins
Hp: haptoglobin
AGP: alpha-1-acid glycoprotein
Cp: ceruloplasmin
ROSs: reactive oxygen species
TNS: tissue necrosis score
OPS: oocyte preservation score
CAT: catalase
MDA: malondialdehyde
